# AFRICAN AMERICAN FAITH LEADERS IN FLINT, MICHIGAN, FACILITATING ACCESS TO SEXUAL HEALTH INFORMATION

**DOI:** 10.1093/geroni/igz038.2298

**Published:** 2019-11-08

**Authors:** Charles R Senteio

**Affiliations:** Rutgers University School of Communication and Information, New Brunswick, New Jersey, United States

## Abstract

In 2006, to respond to the HIV pandemic, the Flint-based YOUR Center collaborated with the National Coalition of Pastors’ Spouses to create an HIV Awareness and Prevention manual for African American faith communities. In 2006 this manual was transformed into Your Blessed Health (YBH), a community-level education and prevention intervention that provides HIV/STI health information to medically underserved communities throughout Michigan. Since then, YBH has trained over 200 faith leaders across 9 denominations in over 80 FBOs across Michigan to help disseminate information concerning the community-level impact of HIV. The YBH impact on congregants and community members has been detailed in peer-reviewed articles and presentations; however, the involvement of the faith leaders themselves has not been described. To address this gap in the literature, we conducted semi-structured interviews with African American faith leaders representing 20+ congregations in Flint who participated in YBH and welcomed the intervention into their congregations. Preliminary results show that faith leaders, whose median age is 65, embraced their role as conduits to health information in the interest of ministering to the “whole person”. They remain committed to educating themselves, and their congregants, to providing health information for various health and wellness issues. Detailing this impact is critical because community-health education and promotion literature suggests that older adult faith leaders can facilitate access to sexual health information through faith-based organizations, and may possess untapped potential as sources of health information across various health conditions. Older adult faith leaders are vital sources of information for medically underserved communities.

